# The effect of tadalafil 5 mg on erection and persistent storage lower urinary tract symptoms after transurethral resection of prostate: a randomized controlled study

**DOI:** 10.1186/s12610-025-00273-2

**Published:** 2025-07-01

**Authors:** Diaa-Eldin Taha, Hossam Nabeeh, Khaled Magdy Zeinelabden, Elsayed Abdelhalim, Salah Elmekawy, Ibrahem Ismail Samaha, Ahmed behiry, Ahmed abd Elmoaty, Tarek Abdelbaky, Ali Ibrahim

**Affiliations:** 1 Department of Urology, Faculty of Medicine, Kafrelsheikh University, Kafrelsheikh University Hospital, El-Giesh Street, Kafrelsheikh, 33155 Egypt; 2 Department of Urology, Faculty of Medicine, Zagazig University, Zagazig, Egypt

**Keywords:** Tadalafil 5 mg, Storage LUTS, Erectile function

## Abstract

**Background:**

We conducted a double-blind, randomized controlled trial to evaluate the effect of early administration of tadalafil (5 mg once daily) on persistent storage lower urinary tract symptoms (LUTS) and erectile function following transurethral resection of the prostate (TURP). All enrolled patients underwent TURP and were randomly assigned to one of two groups: Group A received tadalafil (5 mg once daily) postoperatively, while Group B received a placebo. Erectile function was assessed using the International Index of Erectile Function-5 (IIEF-5) questionnaire, and LUTS were evaluated using the International Prostate Symptom Score (IPSS). The study was conducted at a tertiary care hospital between May 2021 and October 2022.

**Results:**

A total of 195 patients were enrolled and randomized into two groups: Group A (*n* = 103), which received tadalafil 5 mg once daily, and Group B (*n* = 92), which received a placebo. The mean ± standard deviation (SD) scores for the International Index of Erectile Function (IIEF-5) in Group A at 1, 3, and 6 months post-TURP were 11.59 ± 1.90, 19.80 ± 1.12, and 20.97 ± 0.72, respectively. In Group B, the corresponding scores were 4.88 ± 0.82, 12.86 ± 1.56, and 15.32 ± 1.28. These differences were statistically significant (*P* < 0.001).

Similarly, the mean ± SD values for the International Prostate Symptom Score (IPSS) in Group A at 1, 3, and 6 months post-TURP were 7.31 ± 1.66, 4.46 ± 0.97, and 2.33 ± 0.69, respectively, compared to 9.62 ± 3.34, 5.40 ± 1.98, and 2.83 ± 1.27 in Group B (*P* < 0.001).

Regarding storage symptom subscores, Group A demonstrated preoperative and postoperative mean ± SD values of 8.63 ± 1.82, 3.05 ± 0.78, 1.63 ± 0.49, and 0.92 ± 0.67 at preoperative, 1, 3, and 6 months, respectively. In contrast, Group B showed corresponding values of 3.22 ± 0.72, 2.48 ± 0.50, and 1.69 ± 0.47. These differences were also statistically significant (*P* < 0.001).

**Conclusion:**

Tadalafil 5 mg daily as monotherapy following TURP can lead to early improvement in erectile function; however, its effect on persistent storage lower urinary tract symptoms (LUTS) post-TURP appears to be modest.

**Trial Registration:**

Our study has been approved by local ethical committee Kafrelsheikh university(MKSU50-3–1) on 20/5/2021 and by clinical trials (NCT06788704) on 25/1/2025.

## Résumé

**Contexte:**

Nous avons mené un essai contrôlé randomisé en double aveugle pour évaluer l’effet de l’administration précoce de tadalafil (5 mg une fois par jour) sur les symptômes persistants des voies urinaires inférieures (SBAU/LUTS) et la fonction érectile après résection transurétrale de la prostate (RTUP). Tous les patients recrutés ont subi une RTUP et ont été répartis au hasard dans l’un des deux groupes suivants: le groupe A a reçu du tadalafil (5 mg une fois par jour) en postopératoire, tandis que le groupe B a reçu un placebo. La fonction érectile a été évaluée à l’aide du questionnaire de l’International Index of Erectile Function-5 (IIEF-5), et les LUTS ont été évalués à l’aide de l’International Prostate Symptom Score (IPSS). L’étude a été menée dans un hôpital de soins tertiaires entre mai 2021 et octobre 2022.

**Résultats:**

Au total, 195 patients ont été recrutés et répartis aléatoirement en deux groupes: le groupe A (*n* = 103) a reçu 5 mg de tadalafil une fois par jour, et le groupe B (*n* = 92) a reçu un placebo. Les moyennes (± SD) des scores de l’IIEF-5 dans le groupe A à 1, 3 et 6 mois après la RTUP étaient respectivement de 11,59 ± 1,90, 19,80 ± 1,12 et de 20,97 ± 0,72. Dans le groupe B, les scores correspondants étaient de 4,88 ± 0,82, 12,86 ± 1,56 et 15,32 ± 1,28. Ces différences étaient significatives (*P* < 0,001). De même, les valeurs moyennes (± SD) pour l’IPSS dans le groupe A à 1, 3 et 6 mois après la RTUP étaient de 7,31 ± 1,66, 4,46 ± 0,97 et 2,33 ± 0,69, respectivement, contre 9,62 ± 3,34, 5,40 ± 1,98 et 2,83 ± 1,27 dans le groupe B (*P* < 0,001). En ce qui concerne les sous-scores des symptômes de stockage, le groupe A a présenté des valeurs moyennes (± SD) préopératoires et postopératoires de 8,63 ± 1,82, 3,05 ± 0,78, 1,63 ± 0,49 et de 0,92 ± 0,67 à 1, 3 et 6 mois, respectivement. En revanche, le groupe B a montré des valeurs correspondantes de 3,22 ± 0,72, 2,48 ± 0,50 et 1,69 ± 0,47. Ces différences étaient également significatives (*P* < 0,001).

**Conclusion:**

Le tadalafil 5 mg par jour en monothérapie après RTUP peut entraîner une amélioration précoce de la fonction érectile; cependant, son effet sur les symptômes persistants des voies urinaires inférieures (SBAU) après la RTUP semble être modeste.

**Mots-clés:**

Tadalafil 5 mg; LUTS de Stockage; Fonction érectile.

**Enregistrement de l’Essai:**

Notre étude a été approuvée par le comité d’éthique local de l’université Kafrelsheikh (MKSU50-3–1) le 20/5/2021 et par les essais cliniques (NCT06788704) le 25/1/2025.

## Introduction

Men with lower urinary tract symptoms (LUTS) due to benign prostatic hyperplasia (BPH) have a higher incidence of erectile dysfunction (ED) [1]. Moreover, LUTS themselves serve as an independent risk factor for ED, significantly compromising quality of life (QoL). The underlying pathophysiological mechanisms linking LUTS secondary to BPH and ED remain incompletely understood, although both conditions share several common determinants [9, 16].

Transurethral resection of the prostate (TURP) remains the gold standard for managing benign prostatic hyperplasia (BPH), despite the introduction of newer minimally invasive surgical techniques [5, 22]. However, the impact of TURP on sexual function remains uncertain. While some studies report a negative effect, others suggest an improvement in sexual function among patients with ED [19, 26].

TURP may contribute to ED through various mechanisms, primarily due to thermal injury affecting the cavernous nerves during surgery. Additional factors include the psychological impact of the procedure and the temporary cessation of sexual activity in the postoperative period [24].

Previous studies have reported that some patients experience persistent irritative symptoms following TURP, requiring postoperative medication to manage ongoing urinary symptoms. These symptoms significantly impact patients'quality of life after surgery. When selecting candidates for TURP, predicting the persistence of irritative bladder symptoms is crucial; however, studies investigating prognostic factors for bladder irritation remain limited. According to recent research, severe preoperative irritative bladder symptoms or frequent nocturia may increase the likelihood of persistent postoperative symptoms [11].

Phosphodiesterase type 5 inhibitors (PDE5-Is) were initially approved for the treatment of ED; however, they have also been shown to be highly effective in alleviating LUTS in men, regardless of the presence of ED [3, 17, 20, 23]. PDE5-Is promote relaxation of the bladder neck and prostate by increasing nitric oxide levels in smooth muscle, thereby directly influencing micturition phases in addition to their role in penile erection [6, 7]. Another study indicates that a once-daily dose of 5 mg tadalafil is a potentially effective, well-tolerated, and safe treatment option for patients with LUTS [4].

Tadalafil 5 mg once daily is currently approved for the treatment of LUTS, regardless of the presence of coexisting ED [16]. Another study reported that Tadalafil 5 mg once daily improved LUTS in men without ED by a magnitude like that observed in men with ED [2].

Once-daily tadalafil 5 mg has been shown to be most effective in enhancing drug-assisted erectile function in men with ED following radical prostatectomy. It also facilitates early recovery of erectile function post-prostatectomy and may help protect against penile structural changes. However, unassisted erectile function does not improve after discontinuation of active therapy for nine months [18].

Tadalafil monotherapy significantly improves both storage and voiding lower urinary tract symptoms (LUTS) and enhances the International Prostate Symptom Score (IPSS) [12].

To determine the potential benefits of initiating tadalafil early after TURP, we conducted this study to evaluate the efficacy of daily tadalafil 5 mg in managing persistent storage symptoms and improving erectile function post-TURP.

## Materials and Methods

Following approval by the institutional ethics committee, this study was conducted at a tertiary care hospital between May 2021 and October 2022. Patients diagnosed with benign prostatic hyperplasia (BPH) who underwent transurethral resection of the prostate (TURP), either monopolar or bipolar, were eligible for inclusion. Informed consent was obtained from all participants prior to enrollment. Patients were then randomized into two groups: Group A received tadalafil 5 mg once daily, while Group B received a placebo.

All patients included in the study were sexually active prior to undergoing TURP. Patients who were sexually inactive were excluded from analyses related to sexual function, due to the recognized limitations of the International Index of Erectile Function (IIEF-5) in evaluating erectile function among sexually inactive men.

A comprehensive medical history was obtained for each participant. Evaluations included the IIEF-5 questionnaire for erectile function, the International Prostate Symptom Score (IPSS), and the IPSS storage symptom subscore-15 for assessing storage symptoms.

Additional assessments included uroflowmetry, pelvi-abdominal ultrasonography with post-void residual (PVR) urine measurement, and laboratory investigations (urinalysis, urine culture, renal function tests, complete blood count, coagulation profile, and prostate-specific antigen [PSA] level). All evaluations were conducted at baseline (pre-TURP) and at 1, 3, and 6 months postoperatively.

### Randomization

**Fig. 1:**
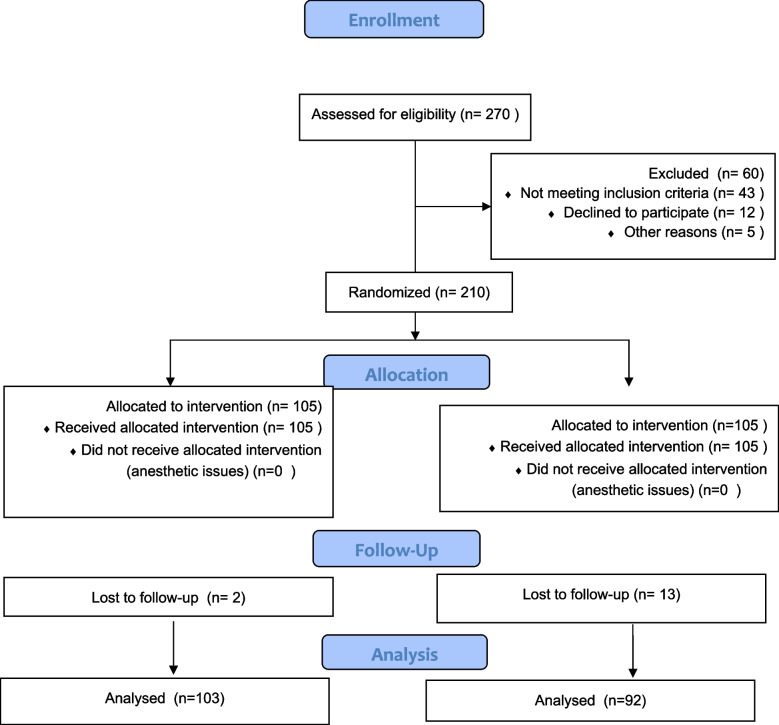
Flowchart illustrating the enrollment, randomization, allocation, follow-up, and analysis of patients in the study. Patients were assessed for eligibility, randomized into two groups (tadalafil 5 mg once daily vs. placebo) after TURP, and followed for six months. The flowchart details participant distribution, loss to follow-up, and final analysis

Patients were randomly assigned in a 1:1 ratio into two groups using simple randomization. Group A received early administration of tadalafil 5 mg once daily starting one week after TURP, while Group B (control group) received a placebo. The randomization process was double-blinded, with both patients and surgeons blinded to group allocation. The study was conducted and will be reported in accordance with the Consolidated Standards of Reporting Trials (CONSORT) guidelines. (Fig. 1).

### Sample size

The sample size was calculated a priori using power analysis (G*Power, version 3.1.9.2). The analysis was conservatively based on three primary outcomes: resolution of storage symptoms post-TURP, improvement in erectile function, and alleviation of lower urinary tract symptoms (LUTS). A Type I error rate (α) of less than 5% and a Type II error rate (β) of less than 20% were assumed. Based on these parameters, a minimum sample size of 105 patients per group was estimated to achieve 80% statistical power, accounting for an anticipated dropout rate of 20%.

### Assessment

The primary outcome of the study was to evaluate the effect of early administration of tadalafil 5 mg once daily on persistent storage lower urinary tract symptoms (LUTS) and erectile function following transurethral resection of the prostate (TURP). The secondary outcome was to assess patient compliance and any adverse effects associated with tadalafil use.

### Statistical analysis

Data analysis was conducted using the Statistical Package for the Social Sciences (SPSS), version 26. Categorical variables were summarized using absolute frequencies and compared using the chi-square test or Fisher’s exact test, as appropriate. Quantitative variables were expressed as means and standard deviations for normally distributed data, or as medians and interquartile ranges for non-normally distributed data. Between-group comparisons of quantitative variables were performed using the independent samples t-test for normally distributed data and the Mann–Whitney U test for non-normally distributed data. Within-group comparisons over two time points were analyzed using the paired samples t-test for normally distributed data and the Wilcoxon signed-rank test for non-normally distributed data. A *p*-value of < 0.05 was considered statistically significant, while a *p*-value of ≤ 0.001 was considered highly significant.

## Results

Of the 270 patients assessed for eligibility, 195 were enrolled in the study. These patients were randomized into two groups: 103 patients (53%) were assigned to Group A, receiving tadalafil 5 mg once daily, and 92 patients (47%) to Group B, receiving a placebo. The allocation process is illustrated in the CONSORT flow diagram Fig. 1**. **

There is a statistically non-significant difference between the studied groups regarding age, BMI, medical comorbidities and prostate size Table 1**.**

**Table 1:** Comparison between the studied groups regarding baseline data

	**Group A**	**Group B**	**t**		**p**	
	**Mean** ± **SD**^**a**^	**Mean ± SD**				
**Age (year)**	63.21 ± 4.78	64.08 ± 3.14	−1.513		0.132	
**BMI**^**b**^** (kg/m**^**2**^**)**	24.01 ± 1.47	24.38 ± 2.24	−1.324		0.188	
	**N = 102 (%)**	**N = 93 (%)**	**χ2**		**p**	
**Diabetes mellitus**	14 (13.7%)	22 (23.7%)	**3.187**		0.074	
**Hypertension**	37 (36.3%)	31 (33.3%)	0.185		0.667	
**Liver disease**	6 (5.9%)	4 (4.3%)	Fisher		0.854	
**Previous surgical history**	68 (66.7%)	63 (67.7%)	0.026		0.873	
**ASA**^**c**^			1.114¥			
**1**	81 (79.4%)	65 (69.9%)				
**2**	15 (14.7%)	22 (23.7%)			0.291	
**3**	5 (4.9%)	6 (6.5%)				
**4**	1 (1%)	0 (0%)				
	Median (IQR)	Median (IQR)		Z		P
**Prostate size**	90(66–120)	90(65–119.5)		−0.184		0.854

This analysis shows that the clinical disparities between the two groups (age, BMI, comorbidities, surgical history and ASA). X^2^ Chi square test t independent sample t test Z Mann Whitney test **p ≤ 0.001 is statistically highly significant *p < 0.05 is statistically significant ¥Chi square for trend test

^a^*SD* Standard deviation

^b^*BMI* Body mass index

^c^*ASA* American society of anesthesiologists

**Fig. 2:**
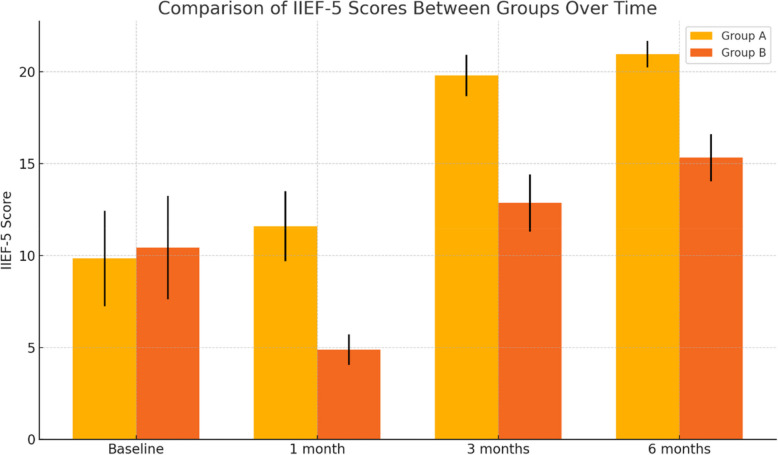
Comparison of International Index of Erectile Function between the study groups before TURP and throughout the 6-month follow-up period

With regard to erectile function, there was no statistically significant difference in baseline IIEF-5 scores between the two groups (*p* = 0.131). In Group A, a significant improvement in IIEF-5 scores was observed following TURP. The mean ± SD IIEF-5 score in Group A was 9.84 ± 2.6 at baseline, which increased to 11.59 ± 1.9, 19.8 ± 1.12, and 20.97 ± 0.72 at 1, 3, and 6 months postoperatively, respectively (*P* < 0.001), as shown in Fig. 2. In contrast, Group B showed a significant decline in erectile function at 1 month post-TURP, with the IIEF-5 score decreasing from a baseline of 10.43 ± 2.81 to 4.88 ± 0.82. A gradual improvement was noted at 3 and 6 months, with scores of 12.86 ± 1.56 and 15.32 ± 1.28, respectively (*P* < 0.001) Table 2**.**

**Table 2:** Comparison between the studied groups regarding IIEF-5 before TURP and during 6 month follow-up period

	**Group A**	**Group B**	**t**	**p**
	**Mean** ± **SD**	**Mean ± SD**		
**IIEF baseline**	9.84 ± 2.6	10.43 ± 2.81	−1.517	0.131
**IIEF 1 month**	11.59 ± 1.9	4.88 ± 0.82	32.488	< 0.001**
**IIEF 3 months**	19.8 ± 1.12	12.86 ± 1.56	35.362	< 0.001**
**IIEF 6 months**	20.97 ± 0.72	15.32 ± 1.28	37.483	< 0.001**

T independent sample t test *p < 0.05 is statistically significant **p ≤ 0.001 is statistically highly significant IIEF: International index of erectile dysfunction

Regarding overall lower urinary tract symptoms (LUTS), there was no statistically significant difference between the groups in terms of baseline International Prostate Symptom Score (IPSS), with a *p*-value of 0.779. Both groups demonstrated a comparable improvement in IPSS, maximum urinary flow rate (Qmax), and post-void residual urine (PVR). However, there was a statistically significant difference between the two groups regarding the storage IPSS score (*p* value: 0.036). as shown in Table 3.

**Table 3:** Comparison between the studied groups regarding IPSS, Storage IPSS, Voiding IPSS, Qmax, and PVR before TURP and during 6 month follow up period

	**Group A**	**Group B**	**t**	***p***
	**Mean** ± **SD**	**Mean ± SD**		
**IPSS**^**a**^** pre**	24.18 ± 2.41	24.08 ± 2.63	0.28	0.779
** IPSS 1 month**	7.31 ± 1.66	9.62 ± 3.34	−6.027	< 0.001**
** IPSS 3 months**	4.46 ± 0.97	5.4 ± 1.98	−4.135	< 0.001**
** IPSS 6 months**	2.33 ± 0.69	2.83 ± 1.27	−3.34	< 0.001**
**STORAGE IPSS pre**	8.63 ± 1.82	8.03 ± 0.22	2.113	0.036*
** STORAGE IPSS 1 month**	3.05 ± 0.78	3.22 ± 0.72	−1.545	< 0.001**
** STORAGE IPSS 3 months**	1.63 ± 0.49	2.48 ± 0.5	−12.077	< 0.001**
** IPSS 6 months**	0.92 ± 0.67	1.69 ± 0.47	−9.19	< 0.001**
**Voiding IPSS pre**	15.55 ± 3.079	16.04 ± 3.445	−1.057	0.292
** Voiding IPSS 1 month**	4.26 ± 1.934	6.41 ± 3.301	−5.466	.000
** Voiding IPSS 3 months**	2.83 ± 1.035	2.95 ± 2.082	-.472	.637
** Voiding IPSS 6 months**	1.41 ± 1.018	1.18 ± 1.259	1.401	.163
**Qmax**^**b**^** pre**	7.54 ± 2.45	8.27 ± 2.28	−3.47	< 0.001**
** Qmax 1 month**	16.05 ± 2.05	15.22 ± 2.0	0.638	< 0.001**
** Qmax 3 months**	20.32 ± 3.15	19.00 ± 2.1	0.897	< 0.001**
** Qmax 6 months**	23.41 ± 3.38	21.56 ± 3.59	5.892	< 0.001**
	**Median (IQR)**	**Median (IQR)**	Z	P
**PVR**^**c**^** pre**	100(90–120)	110(70–160)	−1.041	0.298
** PVR 1 month**	40(30–50)	40(20–50)	−1.096	0.273
** PVR 3 months**	20(20–30)	20(15–30)	−0.956	0.239
** PVR 6 months**	10(10–20)	10(10–20)	−0.772	0.262

T independent sample t test Z Mann Whitney test *p < 0.05 is statistically significant **p ≤ 0.001 is statistically highly significant

^a^*IPSS* International prostate system score

^b^*Qmax* Maximum urine flow rate

^c^*PVR* Post voiding residual

**Fig. 3:**
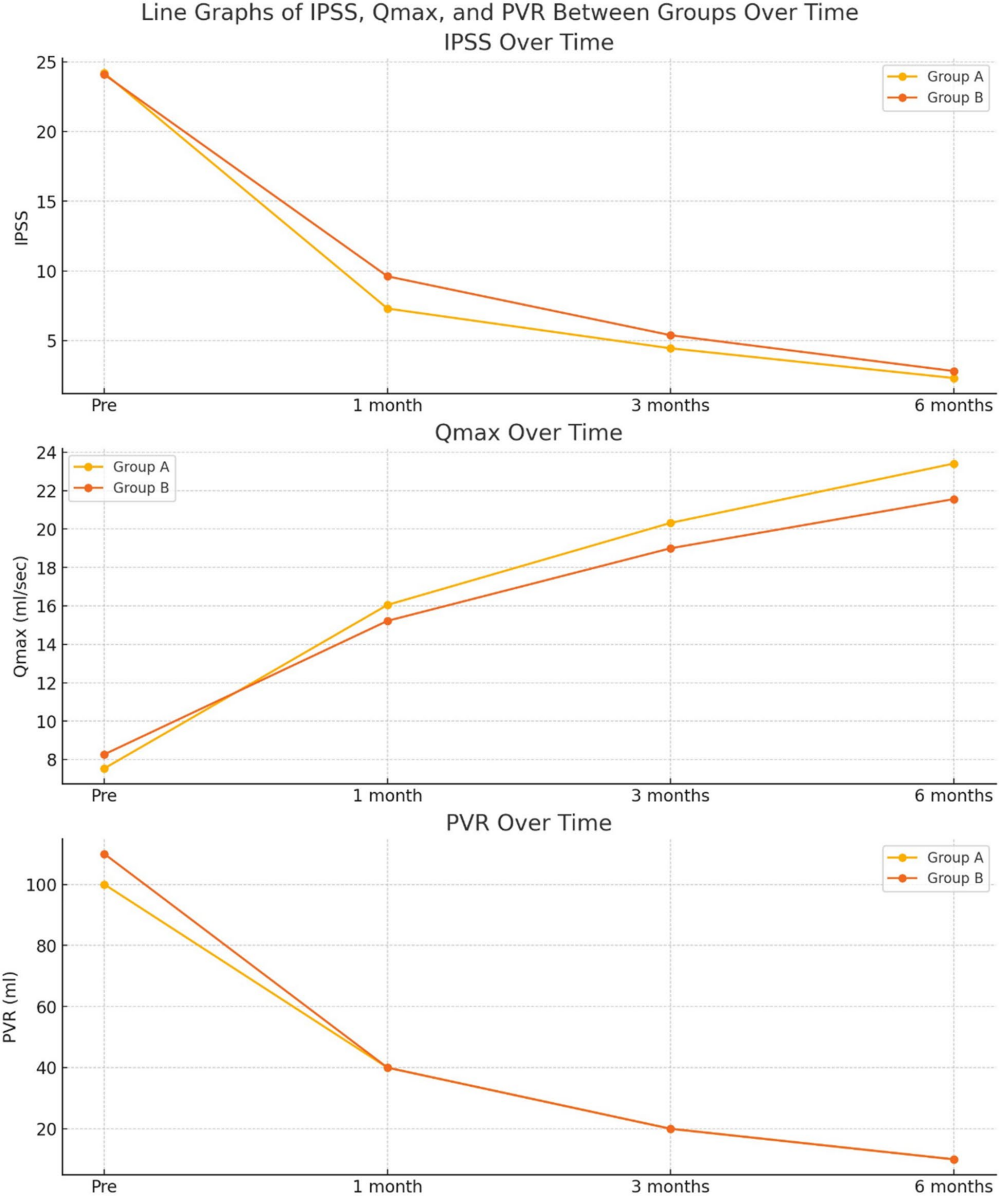
Comparison of International Prostate Symptom Score (IPSS), storage IPSS, maximum urinary flow rate (Qmax), and postvoid residual volume (PVR) between the study groups before TURP and throughout the 6-month follow-up period

The mean (± SD) IPSS in Group A before transurethral resection of the prostate (TURP) was 24.18 ± 2.41, which improved to 7.31 ± 1.66, 4.46 ± 0.97, and 2.33 ± 0.69 at 1, 3, and 6 months post-TURP, respectively (*P* < 0.001). In Group B, the mean (± SD) IPSS before TURP was 24.08 ± 2.63, which improved to 9.62 ± 3.34, 5.4 ± 1.98, and 2.83 ± 1.27 at 1, 3, and 6 months post-TURP, respectively (*P* < 0.001) Fig. 3**.**

The mean (± SD) maximum urinary flow rate (Qmax) in Group A preoperatively was 7.54 ± 2.45, which improved to 16.05 ± 2.05, 20.32 ± 3.15, and 23.41 ± 3.38 at 1, 3, and 6 months postoperatively, respectively (*P* < 0.001). In Group B, the mean (± SD) Qmax preoperatively was 8.27 ± 2.28, and postoperatively it was 15.22 ± 2.0, 19.00 ± 2.1, and 21.56 ± 3.59 at 1, 3, and 6 months, respectively (*P* < 0.001) Fig. 3. 

Both groups demonstrated a significant improvement in post-void residual urine (PVR), with a reduction observed at 1, 3, and 6 months following TURP. However, the difference between the two groups was not statistically significant (*P* = 0.2) Fig. 3**.**

The improvement in the Voiding IPSS score was comparable between the two groups, with no significant difference observed.

A slight improvement in persistent storage symptoms was noted in Group A compared to Group B. The mean (± SD) preoperative storage symptom sub-score in Group A was 8.63 ± 1.82, which improved to 3.05 ± 0.78, 1.63 ± 0.49, and 0.92 ± 0.67 at 1, 3, and 6 months post-TURP, respectively (*P* < 0.001). In Group B, the mean (± SD) preoperative storage symptom sub-score was 8.03 ± 0.22, which improved to 3.22 ± 0.72, 2.48 ± 0.5, and 1.69 ± 0.47 at 1, 3, and 6 months post-TURP, respectively (*P* < 0.001) (Table 3).

Nearly all patients were compliant with daily tadalafil usage. Regarding adverse effects, only 13 patients reported mild headaches and runny noses for a short duration. These symptoms did not result in discontinuation of tadalafil treatment.

## Discussion

PDE5-I treatment was more effective than placebo in patients with ED following nerve-sparing radical prostatectomy, promoting the recovery of erectile function and potentially protecting against penile structural changes [10].

Tadalafil is safe and effective, and can be used as the first-line medication for the clinical treatment on male ED after transurethral resection of prostate [25].

Administration of 5 mg tadalafil once daily resulted in significant improvements in total International Prostate Symptom Score (IPSS), including both voiding and storage subscores, as well as enhancements in IPSS-related quality of life (QoL) and the BPH Impact Index (BII) [4].

Similarly, in our study, and to the best of our knowledge, this is the first trial to compare the effects of early post-TURP administration of tadalafil 5 mg on persistent storage symptoms and erectile function.

In elderly men, ED and LUTS associated with benign prostatic enlargement (BPE) are highly prevalent comorbid conditions that negatively impact quality of life (QoL) and impose a significant economic burden. Preclinical and clinical studies have established that, in addition to aging, various metabolic factors contribute to the onset and progression of both ED and LUTS, leading to penile and nerve alterations, as well as prostate enlargement and inflammation [8]**.**

TURP is widely recognized as the most effective traditional surgical method for treating BPH. Once the bladder is relieved of obstruction, the thickened bladder wall undergoes atrophy, restoring its elasticity and alleviating irritative symptoms. Notably, the degree of symptom improvement is reported to be particularly high in patients with severe preoperative irritative symptoms [14].

However, a recent study indicated that TURP does not alleviate irritative symptoms in all patients, with 20% to 30% requiring postoperative medication due to persistent symptoms, ultimately leading to a decline in quality of life. Several clinical studies have demonstrated significant improvements in LUTS with the use of PDE5 inhibitors. Although the precise mechanism underlying LUTS improvement with PDE5 inhibitors remains unclear, proposed mechanisms include the relaxation of smooth muscle cells in the urogenital tract via the NO/cGMP/PDE5 pathway [13].

In our study, we observed a significant improvement in erectile function in Group A. The mean (± SD) IIEF score in Group A was 9.84 ± 2.6 before TURP, increasing to 11.59 ± 1.9, 19.8 ± 1.12, and 20.97 ± 0.72 at 1, 3, and 6 months post-TURP, respectively (*P* < 0.001).

Regarding overall LUTS, both groups demonstrated comparable improvements in IPSS, Qmax, and PVR. However, in Group A, there was a slight greater improvement in persistent irritative symptoms than Group B. The mean (± SD) preoperative storage symptom sub-score was 8.63 ± 1.82, which decreased to 3.05 ± 0.78, 1.63 ± 0.49, and 0.92 ± 0.67 at 1, 3, and 6 months post-TURP, respectively (P < 0.001). Similarly, in the first systematic review on the use of PDE5 inhibitors for LUTS, Laydner et al. reported that PDE5 inhibitors improve the IPSS score and IIEF-5 but do not significantly affect Qmax [15].

Several randomized controlled trials (RCTs) have demonstrated that PDE5 inhibitors significantly reduce IPSS scores, improving both storage and voiding LUTS, and enhancing patients'quality of life. Similarly, a meta-analysis reported significant improvements in IPSS and IIEF scores with PDE5 inhibitor use, though no significant change was observed in Qmax. Additionally, another study found that tadalafil was both effective and well tolerated in the treatment of erectile dysfunction (ED) and LUTS in sexually active men with both conditions, with significant improvements observed irrespective of baseline severity [21].

### Limitation of study

This study represents the first prospective trial of penile rehabilitation using tadalafil 5 mg once daily post-TURP. However, several limitations were identified in this study. First, the sample size was relatively small, and therefore, a larger-scale study involving a more extensive population is recommended for more robust conclusions.

Additionally, the study did not compare the outcomes of patients who received tadalafil on demand with those who received tadalafil on a daily basis early postoperatively. Also, Further clinical studies with longer follow-up periods are necessary to comprehensively assess the long-term effects of tadalafil on erectile function and irritative symptoms following transurethral resection of the prostate (TURP).

## Conclusion

In comparison to placebo, early Tadalafil 5 mg daily monotherapy after TURP can improve erectile dysfunction. We found that tadalafil 5 mg has a slight improvement in persistent storage symptoms post-TURP.

## Data Availability

The data is contained within the manuscript, any missing details will be available from the corresponding author on reasonable request.

## Abbreviations

LUTS: Lower urinary tract symptoms
TURP: Transurethral resection of the prostate
IPSS: International Prostate Symptom Score
IIEF: International Index of Erectile Function
PDE5-Is: Phosphodiesterase type 5 inhibitors
ED: Erectile dysfunction
BMI: Body Mass Index
RCT: Randomized Controlled Trial
Qmax: Maximum Urine Flow
PVR: Post Voiding Residual
NO: Nitric Oxide
cGMP: Cyclic guanosine monophosphate
BPH: Benign Prostatic Hyperplasia
BPE: Benign Prostatic Enlargement
QoL: Quality Of Life
PSA: Prostatic specific antigen
